# Preterm infant formulas with glucose polymers and reduced lactose content increase the risk of necrotizing enterocolitis

**DOI:** 10.3389/fnut.2026.1768510

**Published:** 2026-05-28

**Authors:** Randal K. Buddington, Scott C. Howard

**Affiliations:** 1 First Breath of Life, Shreveport, LA, United States; 2 Resonance, Memphis, TN, United States; 3 Hospital Sant Joan de Déu Barcelona, Barcelona, Spain

**Keywords:** breast milk, corn syrup solids, glucose polymers, lactose, maltodextrin, necrotizing enterocolitis, preterm formula

## Abstract

After more than 70 years of research, necrotizing enterocolitis (NEC) remains the most serious gastrointestinal disease among preterm infants and a leading cause of mortality, morbidity, and disability. Although causes of NEC are multifactorial, this contribution reviews the causal relationship between commercial preterm formulas (PTF) and NEC. The responses of the immature preterm intestine to PTF and the relationship to NEC have been extensively investigated using animal models and in human studies, but the mechanisms by which PTF increases the risk of NEC have not been fully elucidated. Two categories of risk factors may contribute to the higher rates of NEC with PTF compared to breast milk: (1) protective elements in breast milk are missing from PTF and (2) specific ingredients of PTF increase NEC risk. Our comprehensive review of animal studies and clinical trials highlights at least one substance in each category. The principle carbohydrate in breast milk is non-nocive lactose; in PTF, 50%–60% of the lactose is replaced by glucose polymers, which are not present in breast milk, and represent a pathogenic agent. We advocate a complementary research agenda to identify additional protective elements of breast milk (besides lactose) that could be added to PTF and additional nocive elements of PTF (besides glucose polymers) that could be removed to reduce the risk of NEC.

## Introduction

**FIGURE 1:**
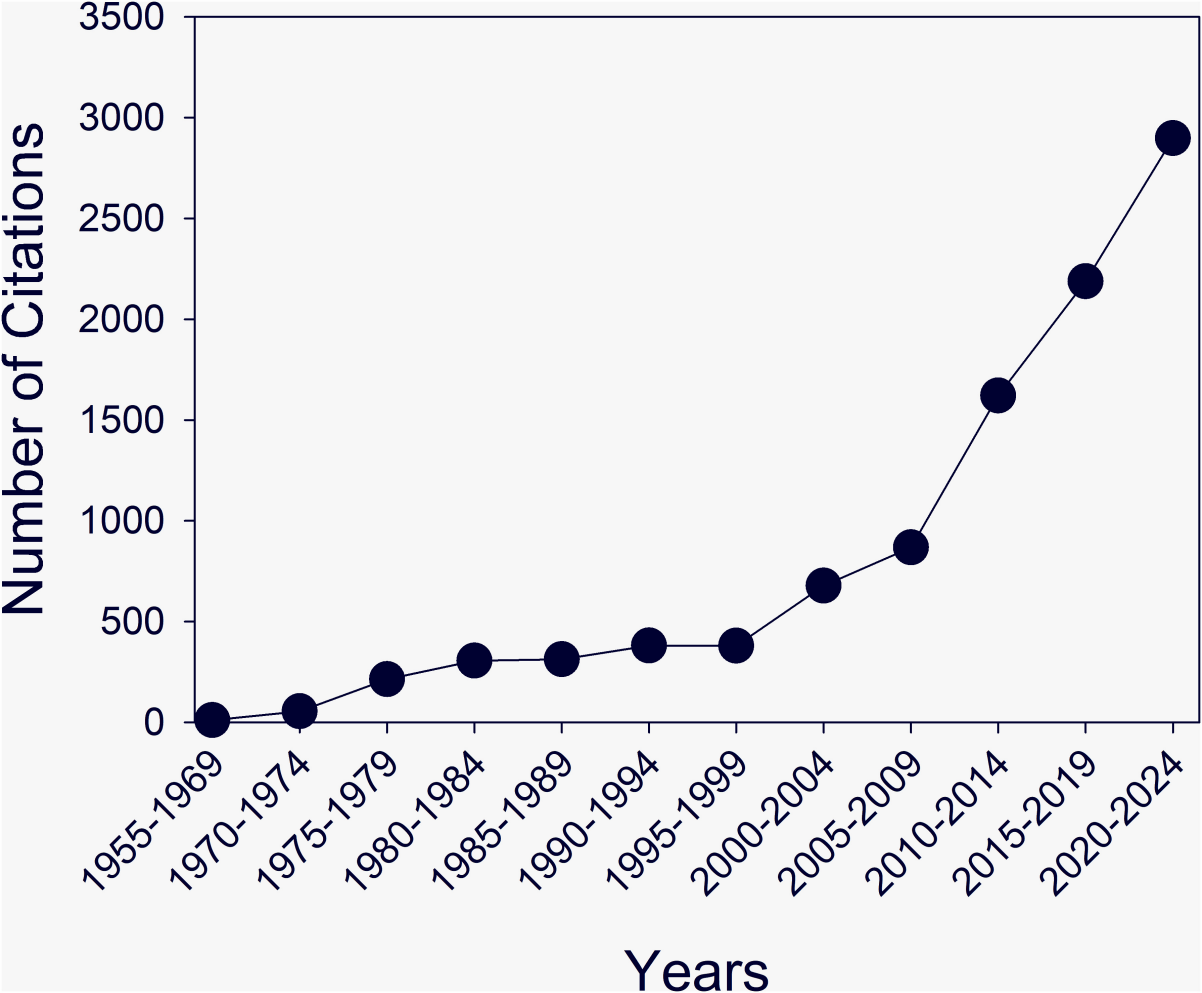
Numbers of publications about necrotizing enterocolitis indexed in PubMed from 1955 to 2024.

Nearly 100 years ago sporadic reports described preterm and low birth weight infants with swollen discolored abdomens, and bloody diarrhea who died with extensive necrosis of the gastrointestinal tract, all of which are hallmarks of necrotizing enterocolitis (NEC). The incidence of NEC was low prior to 1970 when viability was negligible for infants born before 28 weeks or who weighed less than 1,000 g at birth (1). The rapid increase in NEC cases in the 1970s coincided with that improved survival of more extremely low gestational age neonates (ELGAN; born at less than 28 weeks of gestational age), which necessitated increasing use of commercial formulas for nutrition support, since maternal breast milk production is often insufficient in this setting (2). By 1996 survival of infants born at 28 weeks was 90% (3, 4) and has continued to increase, even at earlier gestation ages (5, 6). Unfortunately, the increasing survival rates of ELGAN corresponded with increases in the incidence and severity of NEC (7–11), motivating clinical studies and basic research about NEC (Figure 1).

**FIGURE 2:**
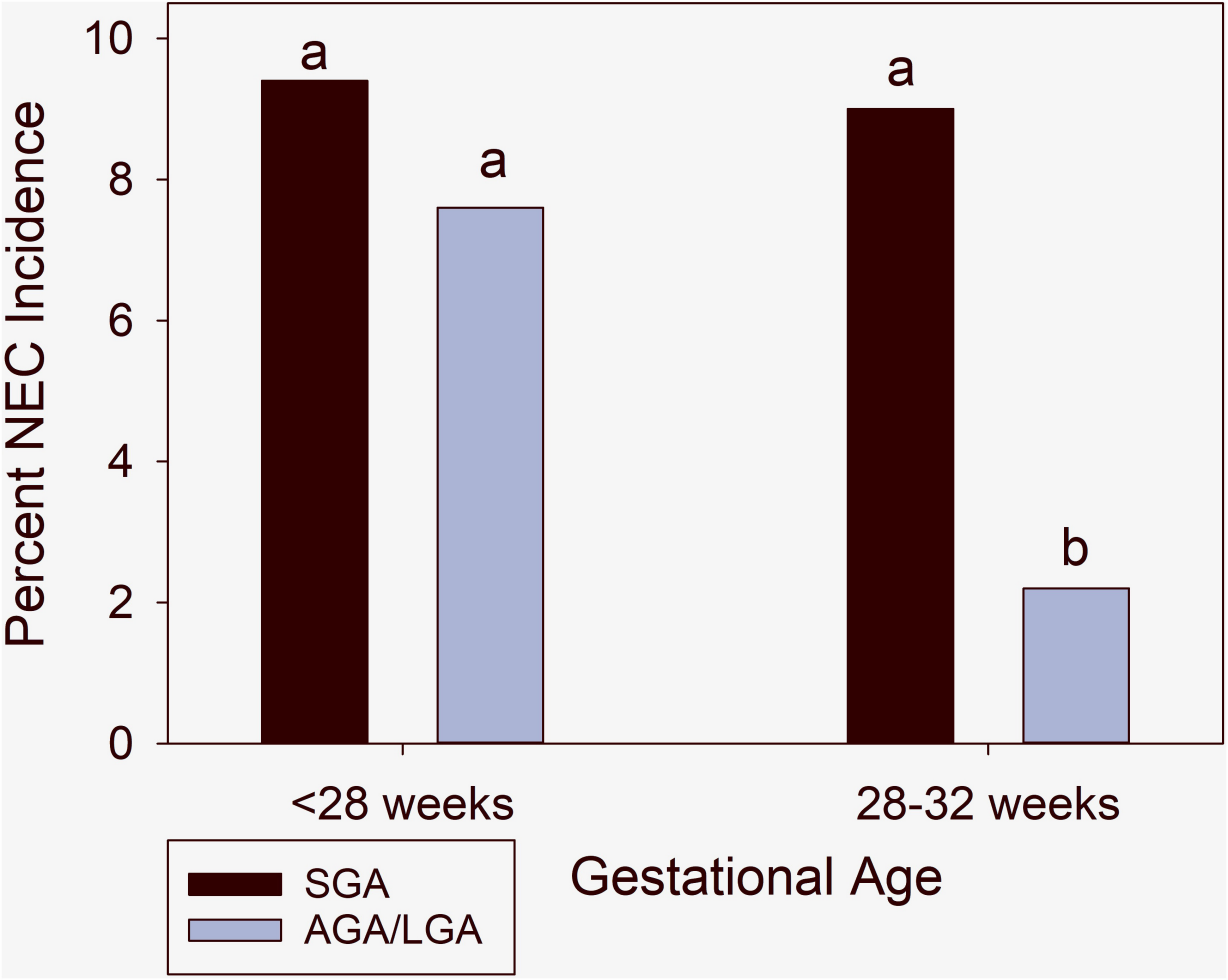
The incidence of necrotizing enterocolitis among infants born at less than 28 weeks and between 28 and 32 weeks and who were either small for gestational age or not. Columns with different letters are significantly different (*P* < 0.05), highlighting the fact that infants born at 28 weeks of gestation or later and who are average or large for gestational age have a 4-fold lower risk of necrotizing enterocolitis. From references (14, 15).

Despite decades of clinical trials, animal research, and cell and tissue-based studies, NEC remains a leading contributor to morbidity and mortality among preterm infants, and especially for those who rely on preterm infant formulas (PTF) for nutrition support (12, 13). ELGAN born earlier in gestation and small for gestational age are at heightened risk (14, 15) (Figure 2).

## Knowledge gaps and scientific controversies

The causal association of PTF with NEC has been elucidated by a deeper understanding of preterm gut development and digestion, refinement of animal studies, and expansion of clinical trials. Recent reviews (16, 17) highlight the importance of protective factors in breast milk but the potential for nocive ingredients in PTF has not been adequately considered. Our comprehensive review provides evidence that the lack of breast milk elements in PTF only partially explains the increased risk of NEC. Findings from animal studies and limited clinical trials reveal the glucose polymers included in PTF at the expense of lactose provides an actionable explanation for the increased risk of NEC associated with PTF. The potential benefits of PTF without glucose polymers need to be evaluated by basic research with animal models and clinical studies with ELGAN at high risk of NEC that include treatment arms of PTF with only lactose.

## History of commercial formulas for preterm infants

Infant formulas have been commercially available for more than 100 years (18–20). Although the benefits of breast milk have been long recognized, the availability of formulas led some to promote “breast to bottle” (21). In the 1950’s formulas were developed specifically to meet the higher nutrient and energy requirements of preterm infants that are not met by breast milk or formulas for term infants. The early PTFs were based on cow’s milk supplemented with protein and included glucose polymers, principally corn syrup and maltodextrin (22), to provide additional energy. The early PTFs resulted in higher growth than breast milk, but were associated with higher NEC incidence, especially among preterm infants born earlier and smaller.

## The causal relationship between PTF and increased risk of NEC

The three major risk factors associated with NEC are prematurity, enteral nutrition using PTF, and disturbance of the gut microbiome (dysbiosis) (13, 23). Numerous other factors contribute to NEC risk (17) which complicate efforts to define the etiology and pathophysiology of NEC. These include transfusions, hypoxic events, hypothermia, other health challenges, and anatomical and physiological defects that reduce perfusion of the gastrointestinal tract. These other factors likely contribute to the 10% of NEC cases that are diagnosed prior to enteral feeding (24).

**FIGURE 3:**
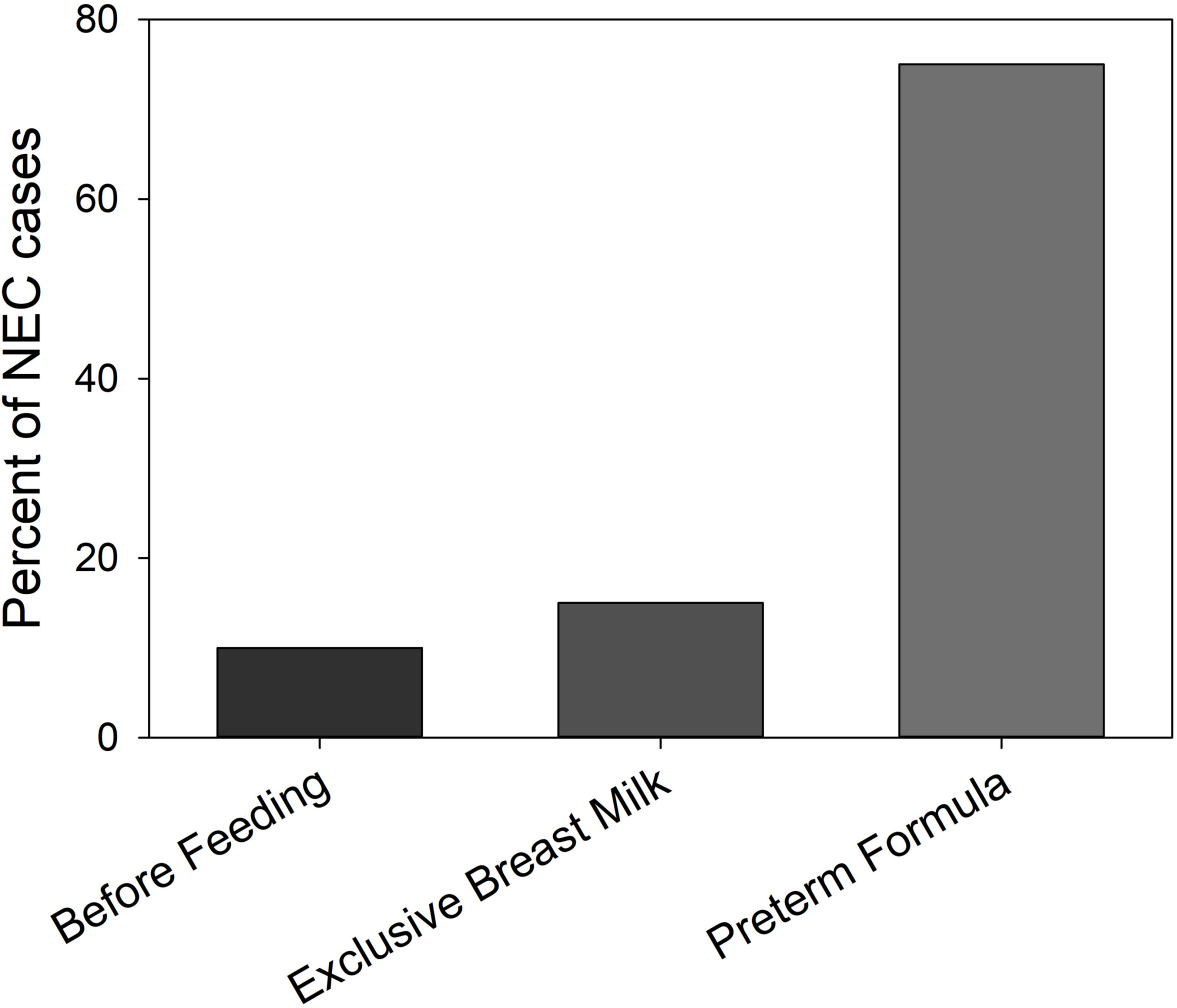
Percentages of necrotizing enterocolitis cases that are diagnosed prior to initiation of enteral nutrition, when only breast milk is fed (mother’s or from donors), or when formula is fed exclusively or as a supplement because of an inadequate supply of breast milk. The figure represents a synthesis of results from several clinical studies.

Providing breast milk is universally recognized as the single most effective clinical approach to reduce NEC among preterm infants, regardless of gestational age, birth weight, and other contributing factors (25). The benefits of breast milk are more pronounced for infants born at earlier gestational ages and small for gestational age (15, 26). The majority of NEC cases (75% or more) occur when enteral nutrition is partly or entirely dependent on PTF because breast milk from the mother or from donors is not available at all or in sufficient quantities (Figure 3). The increased risk of NEC associated with feeding PTF compared with breast milk is consistent among publications and meta-analyses (25, 27, 28). Similarly, the risk of NEC for animal models of prematurity is higher when PTF with glucose polymers is fed compared with suckling the dam or feeding colostrum or milk from other species.

Neonatologists have developed feeding protocols for ELGAN to reduce NEC (29). These include delaying the start of enteral feeds, standardized slow enteral feeding protocols (30) and prolonged parenteral nutrition support, despite its adverse consequences (31), with a gradual replacement by enteral feeds over periods of several days and even weeks, even when breast milk is available (32–34). Feeding is stopped entirely when there are indications of intolerance and possible onset of NEC, generally based on gastric residuals, abdominal distention and redness, presence of bloody stools, temperature instability, and bradycardia. The lengthy delays to attain full enteral feeding rates (120–150 ml/kg-d) reduce NEC when ELGAN are fed commercial PTF but have the disadvantage of slowing growth and presumably development.

When the mother’s own milk is not available or sufficient, supplementing it with donor human milk (DHM) rather than with PTF lowers NEC risk (25, 35–43), despite damage to bioactive elements caused by Holder pasteurization and other processing methods used to assure the safety of DHM (44–47), making this strategy cost-effective (48). When breast milk from the mother or donors is not sufficient, the proportion replaced by PTF predicts the risk of NEC (49–51). For every 10% increase in the amount of human milk replaced by formula the risk of NEC increases by 12%, with a 21% increase in NEC severe enough to require surgery. The greater increase for surgical NEC associated with feeding larger proportions of formula (43, 52, 53) is consistent with greater disease severity, though the finding is not universal (54).

## Complementary theories why PTF increases the risk of NEC

**FIGURE 4:**
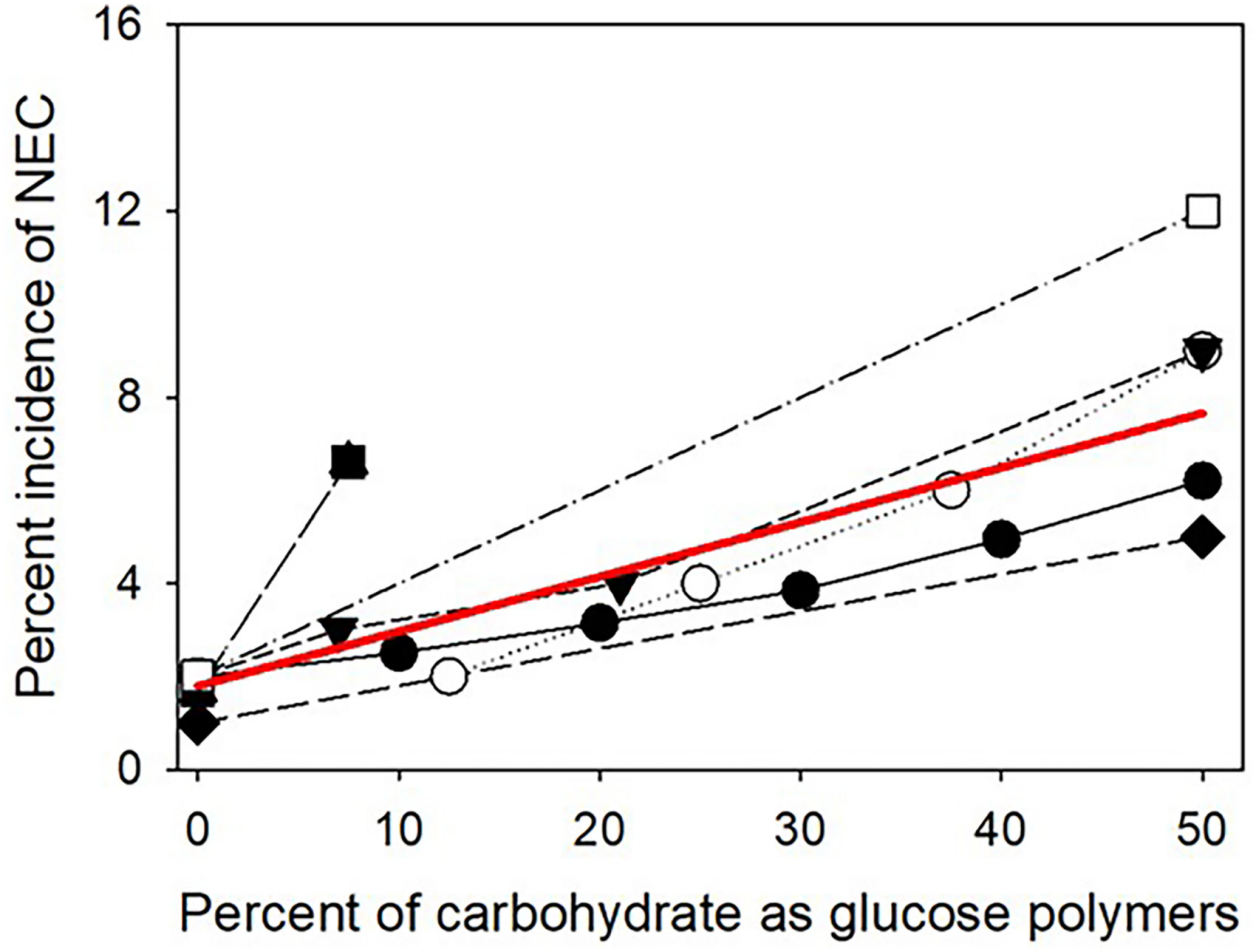
The incidence of necrotizing enterocolitis when preterm infants are exposed to different percentages of glucose polymers based on the dilution of breast milk, mother’s or from donors, using preterm formula. *P* < 0.01 for the linear regression (red line). The different symbols and lines represent findings from references (43, 49, 50, 56, 57).

The causal relationship between PTF and NEC is based on the higher risk of NEC when PTF is fed, but why PTF increases the risk of NEC among preterm infants and in animal models remains uncertain. The increasing risk of NEC associated with diluting breast milk with increasing proportions of PTF (Figure 4) is considered to be the result of the dilution of protective factors in breast milk. This has fostered studies seeking to identify protective components of breast milk that reduce NEC when added to PTF (55). The alternative explanation is increasing the proportion of PTF exposes the immature gut to higher concentrations of nocive substances that are not present in breast milk. To date, only the glucose polymers in PTF have been associated with increased NEC risk, but now that survival of infants born at very early gestational ages is routine, a full re-evaluation of other ingredients is warranted for those born earlier than 28 weeks or earlier than 32 weeks who are small for gestational age and at greatest risk of NEC.

## Preclinical animal models of NEC

Animal models of NEC have been essential to investigate NEC pathogenesis, identify contributing factors, improve prevention and treatment (56–61), and evaluate if novel ingredients, notably elements of breast milk, reduce the risk of NEC associated with PTF (62). Newborn rodents (mice and rats) have been the most common animals used for studies of NEC, beginning in the 1970’s when commercial PTFs were recognized as causing a higher risk of NEC. Induction of NEC in newborn mice and rats typically involves feeding formula with glucose polymers along with exposure to a combination of hypoxia, hypothermia, and inoculation of the gut with bacteria isolated from a patient with NEC (63), but simply feeding formula with glucose polymers is sufficient to induce NEC (64). Like preterm infants fed breast milk, newborn mice and rat pups that suckle the dam are protected from NEC, despite exposure to predisposing hypoxia, hypothermia, and bacterial inoculation.

Preterm pigs have emerged as a relevant species for translational studies (65, 66), and especially for NEC research (67–70). This is because body size and organ anatomy, physiology, and trajectory of development are similar to preterm infants, preterm pigs are compatible with NICU infrastructure and protocols, and as in humans, NEC develops spontaneously by feeding formula with glucose polymers, without the need to induce hypoxia, hypothermia, inoculation with bacteria from a NEC patient, or other non-physiological interventions required to induce NEC in rodent models (69). The onset of NEC symptoms in preterm pigs can occur within 8 h after the start of feeding formula with glucose polymers (71, 72). Preterm pigs with NEC scores of 2 or less often appear clinically normal, with mild (subclinical) NEC that is apparent only at necropsy. The preterm pig model has also been valuable for investigating other factors that contribute to NEC pathogenesis, such as reduced intestinal perfusion (73).

The clinical relevance of animal models has been questioned because the NEC incidence of 50% and even higher greatly exceeds the 5%–10% reported for preterm infants fed PTF. However, an important criterion for the usefulness of preclinical animal models of NEC is a consistently high incidence of NEC to study pathogenesis and for evaluating potential protective and nocive factors. This is accomplished using induction protocols specifically designed to cause high NEC incidence. Enteral nutrition support of the animal models deviates from clinical care of ELGAN by an abrupt start of full enteral nutrition and in most experiments the animals continue to be fed even after they develop evidence of intolerance, resulting in a high incidence of severe NEC.

## Additives that have been investigated for their potential to reduce NEC

Efforts to identify and evaluate components of breast milk that could protect against NEC associated with feeding PTF rely on animal models. Potential protective components include cells, subcellular fractions such as exosomes (74, 75), milk fat globule membranes, other milk extracellular vesicles (76, 77), and various bioactive molecules that include immunoglobulins, milk oligosaccharides, cytokines and chemokines, hormones, and enzymes. Of particular interest are elements of breast milk that influence the developing microbiome (78), inactivate pathogens (79), modulate immune-related mechanisms and signaling pathways implicated in NEC pathogenesis (80, 81), such as TLR4 activation, PPARγ and Wnt/β-catenin mediated signaling (82), enhance intestinal stem cell development (83), protect and restore gastrointestinal tract functions, and contribute to digestion (83–87). Other approaches have investigated probiotics, prebiotics, oligosaccharides, antibiotics, and fecal transplants to improve the developing microbiome and correct dysbiosis. Although probiotics have been evaluated experimentally in clinical trials (88, 89), no commercial PTF currently includes such ingredients meant specifically to reduce NEC. Although providing bovine colostrum and banked human milk to preterm pigs provides protection against NEC (90), clinical evaluations using bovine colostrum-based products, presumably with protective elements, to supplement PTF (91) did not provide preterm infants with significant protection from NEC.

Unfortunately, decades of preclinical studies using animal models to evaluate various novel ingredients for PTF have failed to achieve the level of NEC protection provided to controls that were suckled by the mother, fed bovine colostrum, or breast milk from humans or another species. It is possible that adding protective elements of breast milk and other novel ingredients to PTF does not prevent NEC because this is not sufficient to compensate for the damage caused by glucose polymers in PTF. A limitation of the animal studies that evaluated the responses to the inclusion of breast milk elements and other novel ingredients is that none included a treatment arm with lactose-only PTF that would allow one to distinguish protective factors versus absence of nocive factors, like glucose polymers.

## Why were glucose polymers included in preterm infant formulas?

Carbohydrates are the most abundant macronutrient in mature breast milk at 7% (60–70 g/L) but are lower (20–30 g/L) in colostrum (92, 93) and are comprised mostly (about 80%) of lactose, which is also the base molecule of milk oligosaccharides. In 1911 maltodextrin, known then as dextri-maltose, was approved as an infant feeding product. By 1950 glucose polymers (mainly corn syrup) were added to cow-milk based formulas fed to preterm infants to provide supplemental carbohydrate as an additional source of energy (19). Glucose polymers were included in the first commercial PTF available in the 1970’s and continue to replace 50%–60% of the lactose, despite not being in breast milk.

The decision to partially replace lactose with glucose polymers in PTF was based on the low lactase activity in the intestines of the human fetus and newborn preterm infants, especially those delivered before 32 weeks of gestational age, and concern that preterm infants might be unable to digest the amount of lactose in breast milk (94–106). Fortunately, the low lactase activity of the human fetus (107) increases soon after preterm birth and the onset of feeding (105, 108). Although ELGAN realize increased lactase activity within 5 days after the onset of formula feeding, the magnitude of increase is not as pronounced as that of infants born later in gestation or at term (109). Coinciding with this, an estimated 65% of lactose fed to preterm infants is not digested, based on breath hydrogen as an indicator of bacterial fermentation of the undigested lactose, providing evidence that not all lactose is absorbed (97, 106, 110, 111).

The concern about lactose malabsorption and possible contribution to NEC pathogenesis (112) led to the partial substitution of lactose in PTF with glucose polymers. This decision was supported by early reports that other carbohydrases were expressed by the fetal and preterm human small intestine (107, 113–119). Notably, the higher activities of sucrase, maltase and the associated glucoamylase relative to lactase were considered indicative of the capacity of the preterm infant to digest glucose polymers (120). The enzyme data were corroborated by the slightly higher digestion of glucose polymers than lactose in the upper 20 cm of the small intestine of preterm infants (103) and evidence of enhanced insulin response of infants fed PTF with glucose polymers compared with only lactose (121). Moreover, the lower breath hydrogen of preterm infants fed PTF with a 50:50 blend of lactose and glucose polymers compared with feeding PTF with 100% lactose (99, 101, 122, 123) suggested improved carbohydrate digestion. However, the enzyme and breath hydrogen data have not been confirmed by direct measurements of the amounts of undigested lactose and glucose polymers that enter the colons of preterm infants. Moreover, treating enteral feeds for preterm infants (breast milk or formula) with lactase does not provide significant benefit (124, 125) and any increase in weight gain tends to be transient (126).

Very few of the preterm infants in feeding trials conducted before 2000 that were fed PTF with glucose polymers developed NEC, fostering the perception that glucose polymers were “safe” as ingredients for PTFs. Corresponding with this, the Canadian Pediatric Society, the American Academy of Pediatrics and the European Society of Pediatric Gastroenterology, Hepatology and Nutrition suggested in 1995, 2002 and 2010, respectively (127–129) that glucose polymers should represent about 50% of the carbohydrate in preterm infant formulas, with the balance provided by lactose. This suggestion has remained the industry standard.

## Reconsidering the inclusion of glucose polymers in preterm formulas

Only recently has the potential role of glucose polymers as risk factors of NEC been considered. The majority of studies that evaluated the responses of preterm infants fed PTF with 50%–60% of the carbohydrate as glucose polymers were conducted before 1990 and included few ELGAN because of low survival and exclusionary complications (130). The early evaluations of PTF with glucose polymers also suffered from small sample sizes and design weaknesses, and the majority were funded by formula manufacturers. Moreover, to reduce the risk of NEC, the few ELGAN who were included were often maintained on parenteral nutrition for extended periods of time before beginning a gradual introduction of small volumes of enteral feeds. Findings from early studies have limited relevance to the increasing numbers of ELGAN that are now surviving birth earlier in gestation and at greatest risk of NEC.

Based on evidence reported after 2010, the European Society of Pediatric Gastroenterology, Hepatology and Nutrition recommended the optimal percentage of carbohydrate in PTF represented by glucose polymers needs to be further studied (131). Concerns about PTF with glucose polymers include increased feeding intolerance and risk of NEC, only a limited weight gain advantage, and reduced calcium absorption (131). Compared with breast milk, PTFs with glucose polymers slow gastric emptying (132, 133), cause constipation and harder stools (134–136) that are predictive of impending NEC (137) and correspond with higher laxative use (138). In contrast, PTF with 100% lactose improved stool frequency of preterm infants with insignificant decreases in energy gained from carbohydrate, fat, and protein (98).

## Are glucose polymers digested by preterm infants?

The assumption that glucose polymers are adequately digested by preterm infants has been presumed, not verified. What has been overlooked is the negligible or lack of pancreatic amylase secretion by newborn preterm infants, and even term infants (139–142). Moreover, pancreatic amylase is only marginally inducible after 32 weeks (141, 142), even when preterm infants are fed formula with the glucose polymer corn syrup solids (143) or starch (142). Although salivary amylase is capable of hydrolyzing long chain glucose polymers and partially compensates for the lack of pancreatic amylase (144), the contribution is bypassed with enteral feeding of preterm infants via nasogastric tubes. Amylase is one of the six enzymes that hydrolyze starch and glucose polymers to individual glucose molecules that are absorbed (145, 146). The absence of amylase activity compromises the digestion of glucose polymers (147). The provision of supplemental digestive enzymes to preterm infants has been proposed to compensate for the absence of pancreatic amylase and the reduced secretion of other digestive enzymes (148).

Without amylase and the corresponding luminal hydrolysis of glucose polymers, digestion and absorption of glucose polymers by preterm infants relies entirely on the α-1,4 glycosidic activity and the lower α-1,6 glycosidic activity of glucoamylase associated with the brush border membrane. However, the ability of glucoamylase to hydrolyze glucose polymers decreases as degrees of polymerization increase beyond 6 glucose molecules (149, 150). The glucose polymers used in PTF (e.g., Maltrin M200) have an average degree of polymerization greater than 5, with a significant fraction of polymers having more than 6 glucose molecules. The limitations of glucoamylase are exacerbated by the inhibition of activity by the small glucose polymers that are products of glucose polymer hydrolysis (151). Collectively, the limited hydrolytic activity of glucoamylase for longer chain glucose polymers, inhibition of the enzyme by shorter chain glucose polymers, and the absence of pancreatic (gut) amylase while bypassing salivary amylase via nasogastric feeding limits the ability of preterm infants to digest and absorb the glucose polymers currently included in PTF. Even term infants have limited capacities to digest medium and long chain glucose polymers (152). Hence, an unknown, but likely significant, proportion of the glucose polymers in PTF are not digested.

## Can preterm pigs digest glucose polymers?

The lower activity of glucoamylase in preterm pigs (153) led to questions about the relevance as a model for preterm infants that have higher glucoamylase activity (17). Unlike preterm infants, pancreatic amylase activity is present in preterm pigs, rapidly increases with postnatal age and feeding, even with parenteral nutrition (154), and likely compensates for the lower glucoamylase activity. Because preterm infants lack pancreatic amylase to break down longer-chain glucose polymers, their higher glucoamylase activity does not necessarily confer a greater ability to digest and tolerate glucose polymers than preterm pigs. Direct measurements of glucose polymer digestion during transit of the entire small intestine have not been reported for either preterm infants or preterm pigs.

## Evidence that glucose polymers in PTF increases NEC risk

**FIGURE 5:**
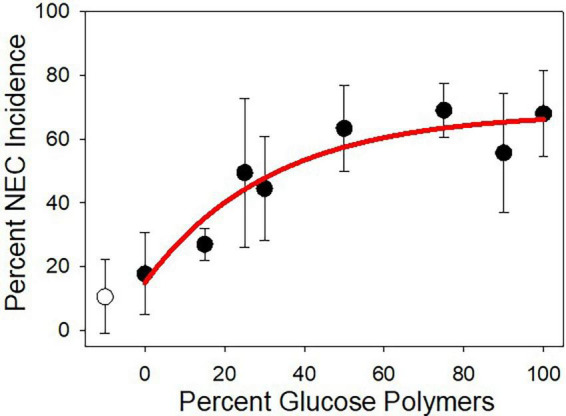
The incidence of necrotizing enterocolitis as a function of the percentage of glucose polymers in formulas fed to preterm pigs. Assembled from references (92, 157–170). The red line is for the nonlinear regression fit of the data (*R* = 0.94, *P* < 0.01). The open circle is the incidence of necrotizing enterocolitis among pigs fed bovine colostrum.

**FIGURE 6:**
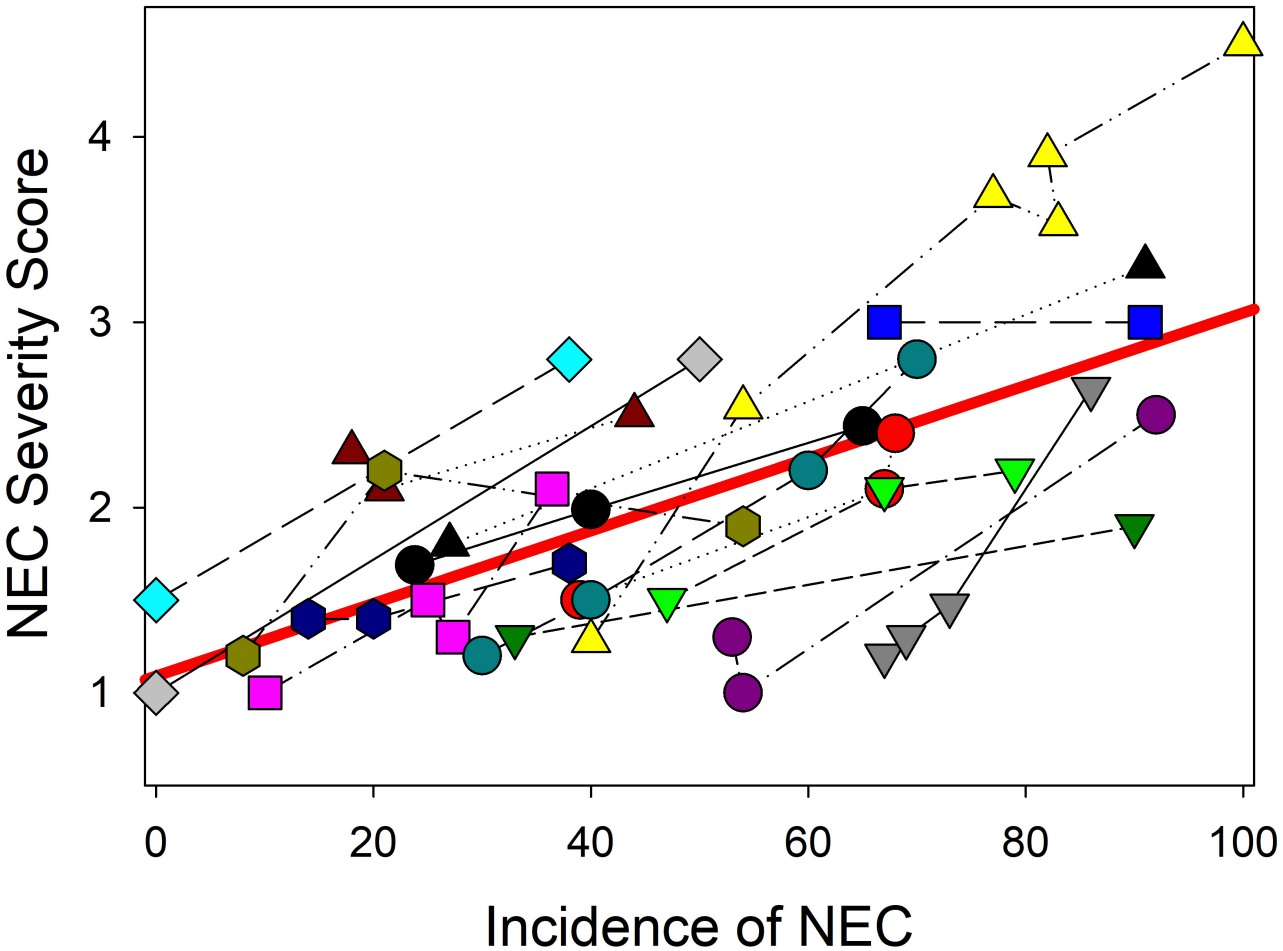
Relationship between the incidence and severity of NEC reported for studies using preterm pigs fed formulas with different percentages of the carbohydrate fraction represented by glucose polymers. The different symbols and lines represent data from references (90, 156, 157, 172, 258–268). The red line is the linear regression for the entire data set; *P* < 0.01.

To date no randomized trial has compared NEC incidence among ELGAN fed PTF with the carbohydrate fraction either 100% lactose, the percentage present in breast milk, with the 50%–60% glucose polymers in commercial PTF. However, a compilation of findings from studies using preterm pigs reveals a dose-response relationship between the incidence of NEC and the proportion of carbohydrate in formula represented by glucose polymers (Figure 5). Formula with as little as 15% of the carbohydrate as glucose polymers increases the risk of NEC relative to formulas with 100% lactose. The higher the percentage of glucose polymers, the greater the risk of NEC up to a maximal risk when glucose polymers comprise 50% or more of carbohydrates. A similar dose-response relationship between glucose polymer and injury to the neonatal intestine was reported for mice (155). For preterm pigs, there is also a direct relationship between the incidence and severity of NEC (Figure 6). It is not understood why maximal risk of NEC in preterm pigs occurs once 50% of the carbohydrates are comprised of glucose polymers without a further increase and if such a maximal risk threshold exists for preterm infants. There is a need to determine if there is a safe level of glucose polymers in PTF.

The absence of protective elements in breast milk does not explain the increased risk of NEC for preterm pigs fed PTF with increasing proportions of glucose polymers. If glucose polymers were safe, the incidence and severity of NEC would be similar for PTF with different proportions of the carbohydrate represented by glucose polymers. Moreover, when preterm pigs are fed breast milk, with all of its potentially protective elements, that is supplemented with glucose polymers they experience NEC at the same rate as when fed PTF with comparable percentages of glucose polymers (156).

The mechanisms of pathogenesis triggered by PTF remain actives area of research. Exposure of the immature gastrointestinal tract to PTF with glucose polymers alters mucosal metabolism and decreases mucin 2 synthesis (157). Sterile organoids derived from 21-weeks human fetuses that were exposed to PTF were smaller, less proliferative, had a higher level of apoptosis, lower differentiation of enteroendocrine cells, and different patterns of gene regulation, particularly for Wnt signaling compared to control organoids cultured with breast milk (158). The organoid data demonstrate that independent of bacterial influences, direct exposure to PTF adversely impacts the immature preterm intestine. Glucose polymers also cause adverse reactions for the mature gastrointestinal tract (145, 159–164).

The findings with preterm pigs are corroborated by a clinical study of 12-days cumulative fluid volume intakes of 99 ELGAN fed PTF with either 99% of the carbohydrate as glucose polymers and 1% lactose or with 33% glucose polymers and 67% lactose (165). The ELGAN were of similar gestational age (26 weeks) and birth weights (815 and 820 g). Enteral feeding was started at 48 h using the two experimental PTF to supplement breast milk. Cumulative fluid volume intake of infants less than 28 weeks was higher when the 67% lactose formula was fed (671 mL/kg birth weight vs. 502). The 67% lactose formula also resulted in 42% higher cumulative fluid intake when breast milk was less than 10% of the fluid intake (586 mL/kg birth weight vs. 339 mL/kg). These findings contradict historical concerns about lactose and feeding intolerance and show that indeed, lactose is a safer carbohydrate than glucose polymers. Importantly, NEC developed in 5 of the 49 (10%) ELGAN fed the low lactose formula, whereas in none of the 50 (0%) fed the high lactose formulas (*P* = 0.027). If glucose polymers were not a risk factor for NEC, the two experimental PTF should have resulted in similar NEC incidences. In a cohort study, NEC developed among healthy newborn preterm infants switched from term formula with only lactose to a preterm formula with glucose polymers (166).

The direct relationship between the amount of glucose polymers in formula fed to ELGAN and NEC incidence corroborates the findings for preterm pigs and neonatal mice and collectively reveal that glucose polymers are a more important risk factor for NEC than the absence of potentially protective elements in breast milk. Indeed, one of the protective elements in breast milk appears to be the presence of lactose (and corresponding absence of glucose polymers). This makes the substitution of lactose with glucose polymers risky and unnecessary.

## Responses of the preterm gut microbiome and metabolome to dietary carbohydrates

Although all aspects of the pathogenesis of NEC have not been elucidated, microbial colonization of the gastrointestinal tract is essential to the disease process. This is evident by the lack of NEC when gnotobiotic (germ-free) rat pups are exposed to an induction protocol that induces NEC in conventional rat pups colonized by bacteria (167). Similarly, germ-free preterm pigs do not develop NEC when fed a maltodextrin-based formula that causes NEC in conventional preterm pigs (69).

The assemblages of colonizing bacteria are highly personalized (168) and have varying influences on the morphological development and proinflammatory responses of the immature gastrointestinal and the risk of NEC (169–171). Importantly, the developing microbiome responds to the type of nutrition support. The gut microbiomes of preterm pigs fed formula with glucose polymers differ from those of litter mates fed human milk or lactose-only PTF, corresponding with different incidences and severities of NEC (172, 173). Similarly, fecal microbiomes of preterm infants differ between those fed PTF with glucose polymers versus breast milk (174, 175), corresponding with different fecal concentrations of short chain fatty acids (176).

Notable similarities shared by preterm pigs and preterm infants exposed to PTF with glucose polymers are the lower growth of bifidobacteria and greater expansion of Enterobacteriaceae, resulting in greater exposure to lipopolysaccharides and other toxins (177–179) that induce the expression of TLR4 and activation of signaling pathways associated with NEC (179). Glucose polymers also adversely influence the mature gut microbiome (180–183). Despite the altered microbiome when formulas with glucose polymers are fed, NEC has not been associated with any specific bacteria (184) or virus (185). Instead, NEC is associated with generalized disturbances in the microbiome, often referred to as dysbiosis (186). This has fostered attempts to “manage” the microbiome of preterm infants using antibiotics, probiotics, prebiotics, and other approaches to reduce the risk or severity of NEC (187–189). Of particular interest are human milk oligosaccharides that influence the infant microbiome (190) and serve other functions (191). Microbiome management by lactose may be another mechanism by which it protects against NEC, since undigested lactose serves as a prebiotic that fosters growth of protective bacteria.

Despite the immature digestive capacities of preterm infants, fecal losses of both lactose and glucose polymers are low in preterm infants (123, 192). This is attributed to fermentation of undigested carbohydrates (97, 110). Metabolites of the resident bacteria, notably short chain fatty acids contribute to gut health and cellular responses (193) and have been considered as biomarkers of NEC pathogenesis (194–196). Preterm infants fed breast milk or commercial PTFs with glucose polymers have different fecal short chain fatty acid profiles (197) that correspond with the different short chain fatty acid profiles between healthy preterm infants and NEC patients (198). Similarly, preterm pigs fed formulas differing only in the source of carbohydrate (lactose versus maltodextrin) have different metabolomic profiles and NEC risk (173). Thus, different metabolomic responses to lactose and glucose polymers are associated with the risk of NEC.

## Reconsidering lactose as the sole source of carbohydrate in preterm formula

The evolution of lactation by mammals is of interest (199, 200), and particularly the origins and biosynthesis of lactose and the milk oligosaccharides (201–203) that are the first and third most abundant components of human breast milk (93, 204, 205) with the amounts and proportions varying from those in the milks of other mammals (206). In human milk, lactose represents 80% of the carbohydrate and the remaining 20% is comprised of about 250 different oligosaccharides (201) and less than 1% fructose (92). Although human milk oligosaccharides share lactose at the terminus, there is wide structural diversity and variation among women in the types that are secreted (207), which in part has a genetic basis (208). The amounts of human milk oligosaccharides in mature breast milk are about 100-fold higher than those present in cow milk (209). Although the level of lactose in breast milk is relatively stable during lactation at about 6 g/dL (210), the levels of human milk oligosaccharides vary widely during lactation (93) with higher amounts in colostrum compared with mature milk. Breast milk composition also differs after giving birth to an infant at term versus preterm (211).

If glucose polymers were a safe and effective source of energy for preterm and term infants and newborns of other mammals, Why did lactation not evolve to secrete glycogen in milk, a glucose polymer synthesized by various tissues? From an evolutionary perspective, it should have been more efficient to include glycogen as the principal carbohydrate in breast milk by adapting the existing metabolic pathway of synthesis used in the liver and other tissues, rather than developing a new metabolic pathway to synthesize lactose, which is only relevant for a single tissue during a limited period of time.

In contrast to the detrimental aspects of glucose polymers, lactose provides multiple benefits that extend from the intestine to systemic, leading to the consideration of lactose as a “bioactive carbohydrate” (212). Undigested lactose influences the structure and functions of colonic microbial communities (213) in a manner consistent with those of a prebiotic (214). The increased proliferation and metabolic activities of health promoting bacteria in response to lactose confer numerous benefits (214–216). The increased breath hydrogen resulting from fermentation of undigested (malabsorbed) lactose may provide benefits to preterm and term infants and should not be confused with hydrogen production associated with disease (96). Furthermore, immunoregulation by lactose induces innate immunity in newborns, including the production and secretion of antimicrobial peptides by the gastrointestinal tract (217). These attributes may have selected for the inability of even term infants to digest the entire dietary loads of lactose associated with breast milk, a finding that has often been considered as lactose intolerance or malabsorption, but instead may be a key adaption to preserve the protective features of lactose.

Evidence suggests that lactose as a critical nutrient for newborns has evolved as a source of galactose (218). In addition to being an energy source, galactose has other critical metabolic and regulatory functions (219, 220) and serves as a structural element of numerous molecules (221). The lower glycemic index of the galactose component of lactose (222) may contribute to the lower risk of subsequent obesity in breastfed infants compared with those fed formula with glucose polymers that have a higher glycemic index (223). The immunoregulatory aspects of milk oligosaccharides are associated with the binding of galactose and lactose moieties of milk oligosaccharides to galectins and other immune-related receptors (224, 225) that inhibit regulatory T-cells (226), regulate mucosal immunity (227), and mediate repair of damaged intestinal epithelium (228), and augments hepatic CD8+ T cell immunity (229). Even mammals that produce milk with little or no lactose (e.g., marsupials, pinnipeds, cetaceans) still provide galactose, generally as an available component of oligosaccharides (230, 231).

The use of glucose polymers for 50%–60% of the carbohydrate in PTF may be depriving preterm infants of the benefits that would have been provided by 100% lactose and the associated galactose, and thus contribute to the increased risk of NEC. The adage “Mother Nature knows best” seems to apply with natural selection favoring breast milk having lactose, not glucose polymers (glycogen), as the primary source of digestible carbohydrate, despite the costs.

There may be concern that PTF with 100% lactose will result in higher osmolarity compared to a combination of lactose and glucose polymers. Maltrin M200, a form of corn syrup solids commonly used in PTF, has an average molecular weight of about 1,000 g/mole with an average degree of polymerization of 5.6, whereas the MW of lactose is 342 g/mole. Therefore, a PTF with 100% lactose as the source of carbohydrate will have about 25% higher osmolality than an otherwise identical PTF with a 50:50 combination of corn syrup solids and lactose. Based on reported osmolalities of commercial PTF ranging from 230 to 320 mOsm/kg, replacing the 50:50 combination with 100% lactose will result in osmolality less than the 450 mOsm/kg, a value considered to be within safe limits, (232) and comparable to that of or less than fortified breast milk at commonly used energy densities (233).

## Identifying other ingredients that may increase the risk of NEC

Intolerance to cow’s milk proteins was initially thought to contribute to NEC (234) but this was not confirmed (235). Hydrolyzing cow’s milk proteins to reduce allergenicity has not reduced formula intolerance or NEC compared to standard PTF with intact proteins (236, 237). Although NEC has not been linked to the use of soy protein in PTF fed to infants allergic to cow’s milk proteins, both cow’s milk and soy protein can cause Food-Protein Induced Enterocolitis Syndrome (FPIES) (238).

The types of fat for PTF remains an active area of research because of implications as both a cause and means to prevent NEC. The accumulation of lipids in intestinal cells has been implicated as contributing to NEC (239–243). In the 1970’s there was a switch from the use of bovine milk fat in PTF to the vegetable oils blends that are currently used. There is interest in returning to the use of bovine milk fat (244) because vegetable oils have different triglyceride structure, lower digestibility, and the greater accumulation in enterocytes than the lipids in breast milk has potential associations with NEC and infant health (240, 245–250). Incorporating lipid in milk fat globule membranes to mimic breast milk increases lipid digestibility *in vitro* (251) and *in vivo* using preterm pigs (252, 253), and reduces infectious illness of late preterm infants fed formula (254). Evidence from neonatal rodents and preterm pigs confirms that predigested fat reduces the risk of NEC and accelerates maturation of the gut (242, 255, 256), though the benefits have not been found in all experiments (257).

## Conclusion

Preterm infant formulas remains a critical component of nutritional support for preterm infants without access to breast milk or when the available volume is not sufficient. The safety of PTF must be improved to reduce the incidence and severity of NEC to rates comparable to those observed when breast milk is fed. This requires identifying and replacing ingredients currently in PTF that increase the risk of NEC (glucose polymers) and adding protective ingredients that mitigate the risk (lactose and perhaps others). Evidence presented here that glucose polymers directly and indirectly damage the immature gastrointestinal tract and trigger NEC via multiple mechanisms justifies additional animal research and independently funded randomized clinical trials using preterm infants at risk of NEC and without access to sufficient volumes of breast milk to determine whether any amount of glucose polymers can be safely included in PTF and the benefits and potential risks of 100% lactose PTF.
